# Modulation of vestibular input by short-term head-down bed rest affects somatosensory perception: implications for space missions

**DOI:** 10.3389/fncir.2023.1197278

**Published:** 2023-07-13

**Authors:** Roberto Gammeri, Adriana Salatino, Maria Pyasik, Emanuele Cirillo, Claudio Zavattaro, Hilary Serra, Lorenzo Pia, Donna R. Roberts, Anna Berti, Raffaella Ricci

**Affiliations:** ^1^Space, Attention and Action (SAN) Lab, Department of Psychology, University of Turin, Turin, Italy; ^2^SpAtial, Motor and Bodily Awareness (SAMBA) Research Group, Department of Psychology, University of Turin, Turin, Italy; ^3^Department of Radiology and Radiological Science, Medical University of South Carolina, Charleston, SC, United States

**Keywords:** vestibular system, sensory attenuation, somatosensory perception, head-down bed rest, tactile perception

## Abstract

**Introduction:**

On Earth, self-produced somatosensory stimuli are typically perceived as less intense than externally generated stimuli of the same intensity, a phenomenon referred to as somatosensory attenuation (SA). Although this phenomenon arises from the integration of multisensory signals, the specific contribution of the vestibular system and the sense of gravity to somatosensory cognition underlying distinction between self-generated and externally generated sensations remains largely unknown. Here, we investigated whether temporary modulation of the gravitational input by head-down tilt bed rest (HDBR)–a well-known Earth-based analog of microgravity—might significantly affect somatosensory perception of self- and externally generated stimuli.

**Methods:**

In this study, 40 healthy participants were tested using short-term HDBR. Participants received a total of 40 non-painful self- and others generated electrical stimuli (20 self- and 20 other-generated stimuli) in an upright and HDBR position while blindfolded. After each stimulus, they were asked to rate the perceived intensity of the stimulation on a Likert scale.

**Results:**

Somatosensory stimulations were perceived as significantly less intense during HDBR compared to upright position, regardless of the agent administering the stimulus. In addition, the magnitude of SA in upright position was negatively correlated with the participants’ somatosensory threshold. Based on the direction of SA in the upright position, participants were divided in two subgroups. In the subgroup experiencing SA, the intensity rating of stimulations generated by others decreased significantly during HDBR, leading to the disappearance of the phenomenon of SA. In the second subgroup, on the other hand, reversed SA was not affected by HDBR.

**Conclusion:**

Modulation of the gravitational input by HDBR produced underestimation of somatosensory stimuli. Furthermore, in participants experiencing SA, the reduction of vestibular inputs by HDBR led to the disappearance of the SA phenomenon. These findings provide new insights into the role of the gravitational input in somatosensory perception and have important implications for astronauts who are exposed to weightlessness during space missions.

## Introduction

Somatosensory processes enable us to detect, localize, and perceive the quality and intensity of sensory stimuli on our bodies, and to distinguish self-generated from externally generated stimuli (Schafer and Marcus, 1973; Blakemore et al., 1998). On Earth, it has been shown that self-produced somatosensory stimuli (i.e., stimuli related to the execution of a voluntary action) are generally perceived as less intense than those externally generated (i.e., stimuli unrelated to one’s own action) of the same intensity (Blakemore et al., 1998; Burin et al., 2017). This phenomenon, named somatosensory attenuation (SA), has been suggested to underlie the distinction between self and non-self, which has a crucial role in detecting and promptly responding to external stimuli that are potentially relevant for survival (Kilteni and Ehrsson, 2017; Pyasik et al., 2021). SA is thought to be rooted in the construction of an internal model, built on the integration of afferent and efferent multisensory signals. Among afferent signals, a relevant role must be played by the vestibular signal which encodes head/body position with respect to gravity. Nevertheless, the specific contribution of the vestibular system and the sense of gravity to somatosensory cognition underlying distinction of self-generated and externally generated sensations is still unknown. To address this issue, we investigated whether temporary modulation of the gravitational input by head-down tilt bed rest (HDBR)–a well-known Earth-based analog of microgravity–might significantly influence perception of a self-generated stimulus produced by one’s own intended movements, compared to an identical externally generated stimulus.

Somatosensory attenuation phenomenon is thought to arise when the sensory consequence of a voluntary action matches the consequence predicted by an internal forward model (Miall and Wolpert, 1996), in which duplicates of the motor commands of voluntary actions are used to predict and suppress the sensory consequences of that specific action (Waszak et al., 2012). In particular, in a self-generated movement, the descending motor command is accompanied by an internal representation of that command, named efference copy, which is then used to predict the sensory feedback of the movement. This sensory prediction is compared with the actual sensory feedback from the sensory receptors or “reafference.” If the prediction matches the actual sensory feedback, sensory attenuation of self-generated stimuli is likely to occur (Blakemore et al., 1998; Borhani et al., 2017; Burin et al., 2017). On Earth, the expectation of the constant force of gravity is an inherent component of this internal model (Carriot et al., 2015). By integrating information from multiple modalities into its internal model, the brain can detect and anticipate the effects of gravity on both self-generated actions and compensatory reflexes (McIntyre et al., 1998; Zupan et al., 2002). As a result, the constructed neural representation of the body and its parts, as well as their movements are normally preserved (Carriot et al., 2015).

In space, the vestibular system is abruptly deprived of the sense of gravity (Demir and Aydın, 2021). This hampered peripheral input may in turn affect vestibular cortical projections to areas where the integration of sensory inputs takes place, such as the parieto-insular cortex, the thalamus, and the temporoparietal cortex (Demertzi et al., 2016; Van Ombergen et al., 2017). Indeed, during spaceflight altered integration of the vestibular input with somatosensory, proprioceptive, and visual signals misinforms the brain with respect to its existing (i.e., Earth-based) internal model of the expected sensory consequences of the movements (Freeman, 2000). The conflict between the brain’s expectation of the sensory feedback and the actual sensory experience is also thought to underlie motion sickness in the early stages of the spaceflight (Carriot et al., 2021). Thus, a correct internal model is crucial to build an adequate representation of our own movements and is fundamental for veridical somatosensory processing of self-generated and externally generated stimuli (Kilteni and Ehrsson, 2020).

In recent years, the SA phenomenon has been widely studied in different sensory modalities, using behavioral and psychophysical methods (Kearney and Brittain, 2021; Kiepe et al., 2021). Some studies suggested the importance of vestibular information both in the construction of a coherent internal model of a movement (Green et al., 2005) and in the modulation of somatosensory perception (Ferrè et al., 2013a,b, 2015; Moro and Harris, 2018), but the specific contribution of a modulation of vestibular signals to the SA phenomenon has never been investigated. Previous studies investigating the effects of temporary postural changes or short period of HDBR of up to 2 h on brain activity reported decreases in EEG power of the alpha, beta, and gamma bands (Schneider et al., 2008; Chang et al., 2011; Spironelli and Angrilli, 2017) and increased cerebral oxygenation in the prefrontal cortex associated with a slight improvement of executive functioning (Mekari et al., 2022). Long-term HDBR is used by space agencies to study changes associated with long-term spaceflight and consists in placing healthy subjects in −6° head-down tilt bed rest. Long-term HDBR is indeed an accepted Earth-based model of the microgravity and represents both physiologically and perceptually the ground position best resembling weightlessness in space environment (Pavy-Le Traon et al., 2007). In these models (Roberts et al., 2010, 2015), as in microgravity (Karmali and Shelhamer, 2008; Clément et al., 2020; Salatino et al., 2021), the weight of vestibular inputs is greatly reduced. However, although the effects of sustained HDBR on different domains of spatial cognition have been investigated (Clément et al., 2008, 2013; Roberts et al., 2019), it is still unclear whether long-term or temporary modulations of vestibular inputs by HDBR may affect the emergence of the SA phenomenon.

With the present study we aimed to investigate whether a modulation of the vestibular signals by short-period HDBR might influence (i) the general perception of somatosensory stimulations and, more specifically, (ii) the intensity rating of self-generated stimuli compared to identical but externally generated stimuli. We hypothesized that short-period HDBR could differentially affect somatosensory perception of self- and externally generated stimuli. Specifically, we expected that HDBR conditions, by reducing the weight of vestibular information, might affect somatosensory perception and the ability to distinguish between self-generated and externally generated sensations as measured by the SA phenomenon.

## Materials and methods

### Participants

Forty healthy volunteers (23 females; age range: 22–27 years old) were recruited for this study. Participants had no history of neurological or psychiatric disease. All participants were classified as right-handed according to the Edinburgh Handedness Inventory (Oldfield, 1971).

All participants gave their written consent after being informed about the experimental procedure of the study, which was approved by the Bioethics Committee of the University of Turin. Participants were volunteers and received no remuneration.

### Sensory attenuation (SA)

During the experiment, the lateral digital nerve of the participants’ dominant index finger was stimulated using 5-mm-diameter Ag/AgCl classical bipolar surface electrodes attached at the lateral side of the tip and base of the finger. The stimulator (Digitimer DS7A) delivered non-painful electrical stimuli. To determine individual somatosensory threshold, participants were instructed to close their eyes and report verbally when they felt stimulation on their right index finger. The threshold was determined by an ascending-descending-ascending staircase method and set at an intensity at which the participant reported feeling a stimulus on 50% of trials (3 out of 6). The stimulation intensity (2.5 times the subjective threshold + 4 mA with 300 V voltage) was the same for each trial and it was chosen according to the results of a preliminary experiment that tested the effect of different intensities (Burin et al., 2017). Two buttons were connected to the electrical stimulator to trigger the stimulation: one was placed under the participant’s index finger and the other one under the experimenter’s index finger [see also (Pyasik et al., 2019)].

Participants were instructed to press the button when they heard “You” (*Self condition*) or to stay still while the experimenter pressed the button when they heard “Me” (*Other condition*). A total of 40 stimuli were administered (20 self-generated and 20 generated by the experimenter). Eight catch trials (i.e., a trial without stimulation) were also included in a random order to avoid response biases and to control for phantom sensations (i.e., false detection of the somatosensory stimuli). The order of the 48 trials was randomized across participants. In order to avoid habituation, every 20 stimulations the experimenter slightly shifted the position of the stimulating electrode. At the end of each trial, participants were asked to rate the perceived intensity of the stimulus (i.e., intensity rating) delivered to their right hand on a 0–7 Likert scale, with 0 indicating “absence of stimulation” and 7 indicating “highest intensity.” Note that participants were instructed that the intensity of the stimuli would never reach the level of pain and that three “familiarization” stimuli were administered by the experimenter before the main experiment to present the participants with the approximate intensity of the stimuli and to avoid disproportionately high ratings for the first stimuli of the main experiment.

### Procedure

**FIGURE 1:**
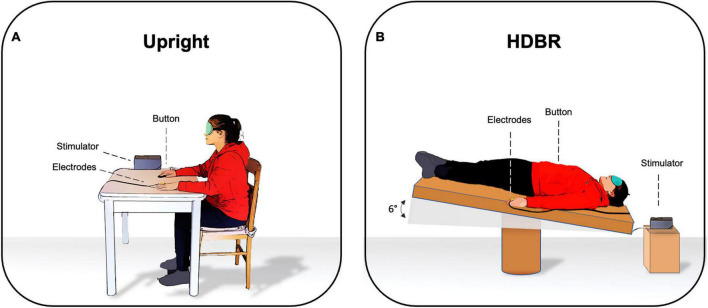
Experimental setting in **(A)** upright position and in **(B)** six degrees head-down tilt bed rest (HDBR).

Participants were blindfolded to avoid the influence of visual cues on somatosensory perception. The SA paradigm was administered under two different experimental conditions according to the position of participants: (1) *Upright*, where participants were seated on a chair and with both arms and hands on the table (2) *HDBR*, where participants were lying supine on the bed with their heads tilted six degrees downward and their arms at their sides (Figure 1). The order of the two conditions was randomized across participants and the somatosensory threshold was calculated twice, i.e., before starting to administer the SA paradigm in each condition. The entire experiment lasted about 1 h; 20 min for each condition with a 10-min break. HDBR was performed in accordance with the international guidelines for the standardization of bed rest studies in the spaceflight context.

### Data analysis

SPSS Statistics software (IBM, version 28.0) was used for data analysis. Self-ranking scores were intra-subject normalized using z-score transformations (i.e., for each participant, each rating value was subtracted by the mean rating and then divided by the standard deviation) in order to obtain comparable measures among the participants (Romano et al., 2014; Burin et al., 2017). The Shapiro-Wilk test, performed on the z-transformed values, indicated that all variables were normally distributed (*p* > 0.05). In order to detect modulations of somatosensory thresholds by HBDR, a paired *t*-test was performed to compare mean values of the two positions. To explore bedrest modulation of sensory attenuation phenomenon, a repeated measures ANOVA with Agent (*Self*, *Other*) and Position (*Upright*, *HDBR*) as within-subject factors was performed on intensity rating. Since SA may not be present in all individuals (Reznik et al., 2015; Burin et al., 2017; Majchrowicz and Wierzchoń, 2021), in order to specifically investigate putative modulation of sensory attenuation by HDBR, we also conducted the same analysis separately in participants who showed sensory attenuation in upright position (i.e., positive difference between other-generated stimuli and self-generated stimuli). *Post hoc* comparisons were performed using the Student–Newman–Keuls test. Correlations between somatosensory thresholds and the amount of sensory attenuation (calculated as the difference between the ratios of Self and Other conditions for each position) were also calculated using Pearson’s correlation. Statistical significance of *p* < 0.05 was assumed.

## Results

### Somatosensory perception

**FIGURE 2:**
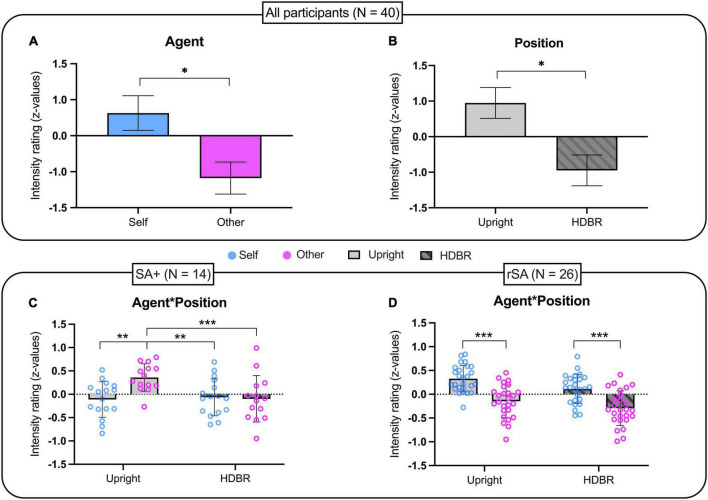
Intensity rating of somatosensory stimulations in: **(A,B)** the whole group (*n* = 40); **(C)** the sub-group of participants showing sensory attenuation in upright position (*n* = 14); **(D)** the sub-group of participants showing reversed sensory attenuation in upright position (*n* = 26). Data have been transformed into z-scores and presented as mean and standard error of the mean (SEM). *p < 0.05, **p < 0.01, ***p ≤ 0.001.

Based on the individual somatosensory threshold, the average stimulation intensity was 8.9 ± 1.24 mA. No statistical difference was found between somatosensory thresholds in the two different positions [t(39) = −0.168; *p* = 0.868]. The repeated-measures ANOVA showed a main effect of Agent [*F*_(1, 39)_ = 6.629; *p* = 0.014; η_p_^2^ = 0.709] and Position [*F*_(1, 39)_ = 4.812; *p* = 0.034; η_p_^2^ = 0.571] while the interaction Agent by Position was not significant [*F*_(1, 39)_ = 1.760; *p* = 0.192; η_p_^2^ = 0.253]. Surprisingly, the significant effect of the factor Agent showed that self-generated stimulations were perceived as more intense than those generated by others (*Self*: Median = 4.5, MAD = 1; *Other*: Median = 4.25, MAD = 1) regardless of the participant’s position (Figure 2A). In addition, the factor position showed that somatosensory stimulation was perceived as more intense during upright than in HDBR condition (*Upright*: Median = 4.75, MAD = 0.75; *HDBR*: Median = 4, MAD = 0.75) regardless of Agent (Figure 2B).

### Sensory attenuation

#### SA + subgroup

In order to investigate putative modulation of sensory suppression phenomenon by HDBR, we selected individuals showing, in the upright position, the sensory attenuation phenomenon (i.e., sensory attenuation for self-generated stimuli). A total of 14 participants (35% of the sample) were identified and their performance as a group was analyzed as before for the entire sample. Also in this group, the *t*-test comparing somatosensory thresholds in the two positions was not significant [t(13) = −0.436; *p* = 0.670]. On the other hand, the repeated measures ANOVA analyzing the effects of Position on sensory attenuation showed a main effect of Agent [*F*_(1, 13)_ = 5.619; *p* = 0.034; η_p_^2^ = 0.592] and a significant interaction Agent by Position [*F*_(1, 13)_ = 9.230; *p* = 0.010; η_p_^2^ = 0.802]. Newman–Keuls *post hoc* analyses showed that while the factor Agent was statistically significant in the Upright condition (*p* = 0.003), it was not significant in the HDBR condition (*p* = 0.907). Specifically, SA attenuation was present in the Upright condition (*Self*: Median = 4, MAD = 1; *Other*: Median = 5, MAD = 1) but not during HDBR (*Self*: Median = 4, MAD = 0.5; *Other*: Median = 4, MAD = 0.75). Moreover, other stimulations in the Upright condition were rated as significantly more intense than those produced by Self (*p* = 0.003) and Other (*p* = 0.001) in HDBR (Figure 2C).

#### rSA subgroup

A total of 26 participants showed reversed sensory attenuation (rSA) at Upright, as self-generated stimulations were rated as more intense than those generated by others. As for previous analyses, no statistical differences of somatosensory threshold were observed between the two positions [t(25) = 0.128; *p* = 0.899]. A repeated measures ANOVA showed an effect of Agent [*F*_(1, 25)_ = 59.058; *p* < 0.001; ηp2 = 0.703], but not Position [*F*_(1, 25)_ = 3.165; *p* = 0.087] nor the interaction of Agent by Position [*F*_(1, 25)_ = 0.395; *p* = 0.536]. More specifically, Self-stimulations were rated as more intense than those produced by Others (*Self*: Median = 5, MAD = 1; *Other*: Median = 4, MAD = 1.25), regardless of the position (Figure 2D).

### Correlation analysis

**FIGURE 3:**
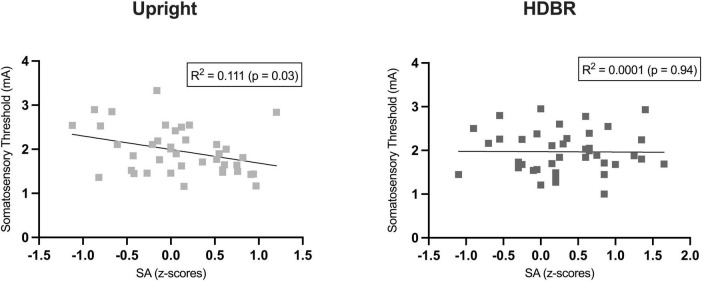
Pearson’s correlation between individual somatosensory thresholds and sensory attenuation (SA) index (i.e., difference between the z-transformed subjective ratings for self-stimulation and other-stimulation).

We computed a series of Pearson correlations in the whole group (*N* = 40) between somatosensory threshold and the sensory attenuation index (i.e., subjective rating for Self-stimulation minus Other-stimulation) for each position (*Upright*, *HDBR*). A significant negative correlation was observed in the Upright condition between the somatosensory threshold and the amount of sensory attenuation (*r* = −0.34, *p* = 0.029). In other words, individuals with lower somatosensory thresholds also had a greater sensory attenuation phenomenon (Figure 3). Interestingly, this correlation was not significant in the HDBR position (*r* = −0.10, *p* = 0.94). No other comparison resulted to be significant.

## Discussion

We investigated the impact of short-period Head-Down Bedrest (HDBR) on the somatosensory perception of self-generated and other-generated stimuli, as measured by the sensory attenuation (SA) phenomenon, whereby self-generated stimuli are perceived as less intense than stimuli generated by others. In all participants, an influence of HDBR on the perception of the intensity of somatosensory stimuli was observed independently of the agent producing the stimulation and in absence of changes of somatosensory threshold. Moreover, a significant modulation of SA by HDBR was found in a subgroup of participants.

### Somatosensory perception

Overall, somatosensory stimuli during HDBR were perceived as less intense than in the upright position, regardless of the agent administering the stimulus. In other words, participants underestimated the intensity of somatosensory stimuli when lying in the head-down position. This change occurred in absence of somatosensory threshold changes, indicating modulation of higher-level somatosensory processes by the HDBR.

To our knowledge no data exist on the putative influence of Earth-based models of microgravity on estimation of the intensity of somatosensory stimuli. However, our results are in line with various experimental studies showing tactile perception modulation induced by vestibular stimulation. For example, left-cold caloric vestibular stimulation (CVS), which activates cortical vestibular regions (Bottini et al., 2005), has been shown to increase tactile sensitivity of both hands in healthy individuals (Ferrè et al., 2011a,b) and improve somatosensory disorders in right (Vallar et al., 1990, 1993) and left brain-damaged patients (Bottini et al., 2005). An improvement of somatosensation has also been induced by subliminal galvanic vestibular stimulation (GVS). In fact, left GVS has been shown to bilaterally increase both tactile sensitivity (Ferrè et al., 2013b) and localization of tactile stimuli (Ferrè et al., 2013c), and both right and left subliminal GVS improved tactile extinction, with lasting effects even after a small number of sessions (Kerkhoff et al., 2011; Schmidt et al., 2013).

On the other hand, our results also seem to be in line with previous studies that have shown that experimental modulations of vestibular input can influence somatosensory processing of noxious stimuli. For example, an overall decrease in pain sensitivity and altered EEG activity of the pain network were observed after 2 h of HDBR (Spironelli and Angrilli, 2011). Notably, the stimulation intensity of our study (i.e., 8.9 ± 1.24 mA) was higher than in previous studies using the same stimulator to investigate non-painful stimuli [3.65 ± 1.09 mA, (Fossataro et al., 2018)] and pain thresholds [4.59 ± 2.44 mA; (Boggio et al., 2008)], but lower than the intensity of stimuli perceived as painful [34.82 ± 10.63 mA, (Fossataro et al., 2018)], suggesting that participants may have perceived the electrical stimulation as moderately painful. In line with these findings, CVS has been found to increase tactile sensitivity but decrease both the perception of pain intensity (Ferrè et al., 2013a) and EEG early cortical responses in somatosensory areas (Ferrè et al., 2015). Consistently, also in clinical populations, CVS has been found to reduce pain perception in patients with central post-stroke pain (Ramachandran et al., 2007; McGeoch et al., 2008, 2009; Spitoni et al., 2016), persistent pain and allodynia (Ngo et al., 2015) and headaches (Wilkinson et al., 2017). However, we did not control for subjective pain experience in our sample, preventing us from drawing firm conclusions on the subjective quality of somatosensory sensations.

The above findings may reflect the complex and multidimensional nature of the somatosensory system, supporting the hypothesis that vestibular signals may have dissociable effects on the various different channels within this system (Ferrè et al., 2015). Pain perception is a complex process that involves the integration of sensory, emotional, and cognitive factors. The perception of painful stimuli is indeed very heterogeneous and may be affected by top-down cognitive processes (Torta et al., 2020), trait personality (Grouper et al., 2021), the intensity of stimulation and anxiety-dependent pain expectancy (Fossataro et al., 2018). Also tactile perception, besides relying on elementary somatosensory processing, involves higher level cognition (Vaishnavi et al., 2000; Ricci et al., 2019, 2021). Thus, it is possible that in our study the vestibular modulation mainly affected high level somatosensory processing (i.e., magnitude estimation of the sensation elicited by electrical stimulation), rather than elementary levels of stimulus processing, as suggested by the unvaried somatosensory threshold during HDBR.

Consistent with the observed behavioral modulation, several neuroimaging investigations over the years have corroborated the evidence of anatomical overlap between vestibular cortical projections and areas involved not only in primary somatosensory processing but also in higher level cognition [for a review see: (Lopez et al., 2012)]. Specifically, fMRI and PET studies in vestibular patients and healthy participants undergoing vestibular stimulation have revealed a distributed vestibular network involving, in addition to the somatosensory cortices, multisensory areas such as the posterior and anterior insula, temporoparietal junction, superior temporal gyrus and the inferior parietal lobule (Lopez and Blanke, 2011; zu Eulenburg et al., 2012). Interestingly, with regard to microgravity analog-models, recent fMRI studies show that HDBR leads to changes in the functional connectivity of vestibular, sensorimotor and somatosensory regions (Cassady et al., 2016). Increased functional connectivity was found between motor and somatosensory areas after long-term HDBR, while decreased functional connectivity was observed in other areas of the vestibular network, such as temporoparietal regions, after both short-term and long-term HDBR (Liao et al., 2015; Cassady et al., 2016). It is important to note that, although these brain areas have been shown to respond differently to intensity-matched tactile and painful stimuli (Su et al., 2019), they responded to both modalities, suggesting that a modulation of their activity may have occurred also in our study. We can hypothesize that, in our study, the temporary reduction of vestibular input by HDBR may have primarily affected the activity of areas involved in higher level processes, such as, for example, magnitude estimation which mainly engages the right posterior parietal cortex (Walsh, 2003; Mennemeier et al., 2005), rather than areas involved in primary somatosensory processing.

In conclusion, our results provide evidence that short-term HDBR induces a general subjective underestimation of the intensity of somatosensory stimuli. Although there are several lines of evidence supporting the hypothesis that this effect can be attributed to decreased vestibular afferents and altered activity within regions contributing to somatosensory cognition, the present study does not directly assess the neural correlates of behavioral changes. Future studies are needed to investigate the neural mechanisms underlying the observed effects.

### Sensory attenuation (SA)

Unexpectedly, in our sample, only 35% of the participants showed, at individual level, *sensory attenuation* for self-produced stimuli in the upright position (SA +), while the other participants showed *reversed SA* (rSA), i.e., self-generated stimuli were rated as more intense than those generated by others (Reznik et al., 2015; Majchrowicz and Wierzchoń, 2021). Interestingly, SA + was modulated by HDBR while rSA was not, suggesting that different processes may be activated.

According to Reznik and collaborators (Reznik et al., 2015), the magnitude and the direction of SA phenomena may depend on the intensity of stimulation, as SA + would occur when active self-generated actions result in supra-threshold stimuli. Here, we only used supra-threshold stimuli but rSA was found in the majority of the participants, therefore other factors need to be considered. For example, other studies suggest that the amplitude of SA is modulated also by the action-effect contingency [i.e., the temporal proximity between actions and their sensory consequences, (Baess et al., 2011; Dogge et al., 2019; Han et al., 2022)] or the strength of the agent’s prior beliefs (Desantis et al., 2012). In our study the contingency and the predictability of the outcomes were kept constant across conditions, while the participants’ beliefs were not controlled.

Interestingly, our data suggest that the individual somatosensory threshold may play a relevant role in the sensory attenuation of self-generated stimuli. Indeed, in the upright position, a negative correlation was found between somatosensory thresholds and SA scores, indexing that lower somatosensory thresholds facilitate the emergence of the SA phenomenon. Furthermore, empirical evidence suggests that both somatosensory perception and the extent of SA are significantly modulated by the subjective feeling of body ownership (Pia et al., 2013; Kilteni and Ehrsson, 2017; Burin et al., 2018; Pyasik et al., 2019, 2021; Ataka et al., 2022). Specifically, a decrease in somatosensory sensitivity has been linked to increased hand-disownership (Ataka et al., 2022) and a greater sense of ownership over one’s body leads to greater sensory attenuation phenomenon (Kilteni and Ehrsson, 2017). Consequently, we speculate that the observed individual heterogeneity of SA scores may be attributed to individual differences in somatosensory threshold, which could indicate higher body ownership (BO) in SA + group compared to rSA.

Consistently, a different modulation of the subjective intensity for self- and external-generated stimulations was observed in the two groups. Indeed, in the SA + group the intensity rating of stimuli generated by others strongly decreased in the HDBR position, leading to the disappearance of the SA phenomenon. On the contrary, the reversed SA (rSA) observed in the majority of participants was not modulated by HDBR. As previously discussed, SA + group demonstrated higher SA magnitude and lower somatosensory threshold, while the rSA group showed reversed sensory attenuation and higher somatosensory threshold. Interestingly, previous evidence also suggests that an alteration of vestibular inputs can result in a decreased sense of BO and in a reduced reliability in external references during tactile localization (Pavlidou et al., 2018; Ponzo et al., 2018; Unwalla et al., 2021; Gammeri et al., 2022).

Thus, if SA + group is more sensitive to bodily information as suggested by the lower somatosensory threshold, the observed disappearance of SA during HDBR may be attributed to the reduction of vestibular input generated by the head-down tilt position. In contrast, if the rSA group rely less on bodily information as suggested by the higher somatosensory threshold, the reversed sensory attenuation may be not affected by the vestibular signals’ reduction. These interpretations support the hypothesis that vestibular signals play a key role in self-other distinction (Deroualle and Lopez, 2014; Lenggenhager and Lopez, 2015; Lopez et al., 2015), suggesting that in simulated microgravity the boundaries between self- and externally generated stimuli can be lost. Further investigations should explore the relationship between somatosensory perception and body ownership, as well as its interaction with the vestibular system, in order to elucidate the mechanisms underlying the disappearance of sensory attenuation in the HDBR-other stimulations condition.

### Implications for space research

Taken together, these findings suggest that modulation of vestibular input by short-period HDBR has an impact on how we process somatosensory information, particularly when sensory attenuation occurs. In space, the neurosensory response to microgravity leads to complex disorientation and motion sickness [i.e., Space Adaptation Syndrome and Space Motion Sickness; (Clément and Reschke, 2008; Wood et al., 2011; Clément et al., 2013)] in the early stages of spaceflight. Within a few days, most sensorimotor impairments resolve, but may reappear upon return to Earth, both after long and shorter space missions (Reschke, 1990; Paloski et al., 1993; Wood et al., 2015; Reschke et al., 2018). Importantly, subtle disturbances in somatosensory cognition may still be present in the later stages of the spaceflight, which, if unrecognized, could significantly impair the crew performance. In particular, during space missions, altered somatosensory perception of externally generated stimuli due to reduced vestibular inputs could result in reduced perception of one’s body boundaries, affecting dexterity, motor performance, and ultimately increasing the risk of accidents and errors during critical operations. Given the technical limitation of medical interventions in space environments, undetected somatosensory signaling impairment in astronauts could delay the detection of illnesses and interfere with ambitious long-term space missions. Although the evidence on how microgravity or simulated microgravity might affect somatosensory functions is still scant and controversial, our data may provide new insight into the putative effects of microgravity on somatosensory cognition of self- and other-generated stimuli.

## Limitations and future directions

Despite the interesting findings reported in this study, there are several limitations that must be acknowledged. Firstly, most of the published studies on the effects of vestibular input modulation on somatosensory processing have been conducted using different techniques other than microgravity or simulated microgravity, which might affect the vestibular system in different ways. In addition, most HDBR studies have used long-term protocols, lasting more than 7 days, in sharp contrast to our study, which employed a short-term protocol lasting approximately 30 min. Although there is evidence suggesting that electrocortical activity is relatively unaffected by protocol duration (Brauns et al., 2021), it is worth noting that no prior research has specifically examined the influence of HDBR duration on somatosensory perception using behavioral tasks. Therefore, to validate the current findings and support their generalization to spaceflight conditions, future long-term HDBR studies need to be conducted. Secondly, we did not control for the individual degree of body ownership and explicit pain perception, which may have influenced the observed outcomes. In fact, although there is evidence on the relationship between somatosensory threshold, the extent of body ownership, somatosensory sensation, and the magnitude of sensory attenuation, this interaction was not controlled for in the current study. Furthermore, it is crucial to consider that in our study all participants were blindfolded and we cannot dismiss the possibility of an additional effect resulting from visual deprivation. On one hand, previous research indicates that the absence of vision may have an impact on somatosensory perception, generating increased activation of vestibular and somatosensory areas (Marx et al., 2003, 2004). On the other hand, the enrichment of multisensory processing by adding visual information, may facilitate the distinction between self-generated stimuli and stimuli generated by others. Consequently, the precise extent to which these variables influenced the observed outcomes remains to be determined. Finally, the lack of neuroimaging data to support our interpretations is another limitation of this study. Although we interpreted the observed modulation of somatosensory perception in response to HDBR based on prior neurophysiological evidence, it is crucial to emphasize that the existing evidence comes from studies employing techniques and protocols different from those used in this specific investigation. Future research should aim to address these limitations and provide more comprehensive insights into the neurofunctional mechanisms underlying the modulation of somatosensory processing in microgravity environments.

## Data availability statement

The raw data supporting the conclusions of this article will be made available by the authors, without undue reservation.

## Ethics statement

The studies involving human participants were reviewed and approved by the Bioethics Committee of the University of Turin. The patients/participants provided their written informed consent to participate in this study.

## Author contributions

RG: investigation, data curation, formal analysis, writing, reviewing, and visualization. AS and MP: definition, conceptualization, methodology, resources, software, and validation. EC, CZ, and HS: writing, reviewing, and visualization. LP, DR, and AB: writing and reviewing. RR: definition, conceptualization, writing and reviewing, supervision, and project administration. All authors contributed to the article and approved the submitted version.
